# Practical Anatomy

**Published:** 1886-08

**Authors:** 


Practical Anatomy. A Manual of Dissections. *By* Christopher Heath, f. r. c. s.; *Professor of Clinical Surgery in the University College, London, etc., etc. Revised by* Rickman J. Godlee, m. s., *London,* f. r. c. s. *Octavo vol., pp. 567, Illustrated. Philadelphia:* P. Blakiston, Son & Co.

Professor Heath's work has reached its sixth edition. It is a trifle enlarged over preceding editions, and has fourteen beautifully colored plates representing practical dissections,—the arteries and veins being stained red and blue, respectively. The wood-cuts throughout the entire book are quite satis-factory, showing the finer lines more distinctly than is usual in works of this character. The reviewer mentions this fact because of its importance to the young student of medicine, to whom a correct picture often "talks clearer than words." The directions for making a dissection are explicit and to the point. The advice of the author as to the "method of study" should be heeded by all dissectors. The descriptions are accurate and the book fully sustains its past reputation as a practical working guide for the student. F. C. S.

